# Case in Which the Bones of a Fœtus Were Passed through an Opening in the Abdomen, by Which Aperture the Catamenia Afterwards Flowed

**Published:** 1827-10

**Authors:** 

**Affiliations:** St. George's Hospital


### EXTRA-UTERINE FŒTATION.

*Case in which the Bones of a Fœtus were passed through an Opening in the Abdomen, by which aperture the Catamenia afterwards flowed.* Treated at St. George's Hospital, by Mr. Gunning.

March 5th, 1823.—Elizabeth Allerwell, ætat. twenty-eight, was married twelve years ago, and had a child four years after, born in the eighth month of pregnancy, which only lived two days. Her medical attendant told her she was made "very strait," on which account it would be well for her not to become pregnant again. Four years after she again fell with child, and expected to be confined in July 1820, but, at the end of the seventh month of her reckoning, the breasts became flaccid, and (to use her own expression) she "lost life," the child having previously been very lively. She did not menstruate during the first seven months, but at this time a discharge apparently menstrual came on, and has returned at regular periods ever since; so that the catamenia appeared at the end of the seventh, eighth, and ninth months of this last pregnancy. For the first seven months she was very sick, and otherwise out of health, but during the last two months she felt much better, and did not increase in size. At the time she expected to be confined, she had labour-pains, returning at regular intervals, and in all respects corresponding to what she had experienced during her first confinement, except that the child did not descend into the pelvis. The pains ceased at the end of the third day, when her health improved; menstruation came on at the end of the month, and she continued free from complaint till June 1821, the tumor in the abdomen remaining exactly of the same size.

At this period she was suddenly seized, while engaged in haymaking, with twisting pain in the tumor, which became so violent as to induce her to come to St. George's Hospital in July, where she continued till September of the same year. The treatment consisted chiefly in the application of leeches, and other means calculated to alleviate the pain.

In the following May (1822,) she was again admitted into the hospital for a few weeks, the centre of the tumor having become dark-coloured, with return of pain. A small quantity of yellow matter discharged itself through the umbilicus, but the opening presently closed again. A second opening took place two inches below, which has continued ever since to discharge in varying quantity; but no collection of matter, as in an abscess, ever appears to have formed. The pain has diminished since the swelling burst, but has always existed in some degree, particularly about the right iliac region. Her health has been tolerably good, so that she has never been prevented from taking exercise till the present time; but, when she stands up, she feels the tumor press towards the opening, and she has a good deal of bearing down in voiding her urine. The swelling is of considerable size, of different degrees of hardness, and irregular in shape, although it approaches to that of a circle. The skin is attached to it, and is of a dark red colour for some extent round the orifice. Menstruation is rather copious, but not painful.

February 1823.—About a month ago the discharge became dark-coloured and offensive, and a portion of bone could be felt projecting. A small opening was made (March 12th,) with a probe-pointed bistoury, sufficient to extract this, which was evidently the transverse process of a vertebra. Next day another small portion of bone was removed, of the same shape and appearance. Sponge tent was introduced once or twice, but gave her considerable pain. By the end of the month, nine pieces of bone, of the same size, had been extracted.

April 20th.—No bone has been detached for the last three weeks, though some can be felt with the probe. Her health continues pretty good, but she has become rather thinner since her admission. She is allowed nourishing diet, with wine, &c.

May 23d.—Within the last three weeks, considerable progress has been made: the bone so long felt adhering to the soft parts proved to be one of the ribs, which was extracted entire, since which time several more have been removed, making in all thirteen pieces of the vertebræ, ten ribs, and one entire scapula. Several portions of the soft parts of the fœtus have also been taken out. All the bones look as if macerated, the cartilages being quite absorbed. Three days ago, two small incisions were made with a bistoury, to allow of the extraction of the scapula, and slight dilatation has been made with Weiss's dilator. The wound is dressed with oil and lint, and a poultice over it. She does not suffer from the operation, so that, for the last week, some portions of bone have been removed every day.

June 13th.—Some irritation has been excited latterly by the bones of the head, which have stuck in the wound. A small opening has been made, to allow the occipital bone to pass; after which the others were removed without much difficulty.

20th.—The tumor has diminished considerably since the extraction of the head, and she is now free from pain. There is a good deal of discharge, but it is easily evacuated by turning her upon the face twice a day. Nearly the whole skeleton has now come away; the parts which remain are principally the bones of the hands and feet.

September 15th.—All the bones which remained at the date of last report were removed by the beginning of August, and the whole were presented by Mr. Gunning to the College of Surgeons. She has gained flesh, and become strong and active,—assisting the nurses: a slight discharge (about one or two teaspoonsful in the course of the day,) continues to ooze from the opening. The tumor is very much diminished in size. A fortnight ago, after five months' suppression of the catamenia, a discharge resembling the menses appeared at the wound. The catamenia have again returned, per vaginam, and are now present, while a discharge apparently menstrual also takes place from the wound. An examination was made per vaginam, and the uterus appeared to be in its natural situation, the os uteri of its usual size: the tumor could be felt on the left side, pressing on the vagina.

October 1st.—Menstruation has again returned, and, as before, the discharge passes partly by the abdominal aperture.

1824. March 24th.—She went out a short time ago, at her own desire, with a fistulous opening still remaining. There has been very little discharge, but two very minute portions of bone have been evacuated.

1825. July 21st.—She calls at the hospital occasionally; is much improved in health; the wound is not healed, and the catamenia continue to flow from it at the monthly periods.

1826. April 4th.—Continues in the same state, menstruating regularly, the discharge flowing partly per vaginam, and partly by the opening in the abdomen.

